# Exploring the mechanism of avenanthramide in the treatment of atherosclerosis based on network pharmacology and molecular docking: An observational study

**DOI:** 10.1097/MD.0000000000040932

**Published:** 2024-12-20

**Authors:** Zhigang Wang, Longzhi Fang, Meng Han, Kangzhe Liu, Yuanmei Zheng, Yibei Zhan

**Affiliations:** a Hubei Key Laboratory for Kidney Disease Pathogenesis and Intervention, School of Medicine, Hubei Polytechnic University, Hubei, China; b College of Chemistry and Chemical Engineering, Hubei Polytechnic University, Huangshi, Hubei, China; c Hubei Key Laboratory of Mine Environmental Pollution Control & Remediation, Hubei Polytechnic University, Hubei, China.

**Keywords:** anti-inflammatory, atherosclerosis, avenanthramide, molecular docking, network pharmacology

## Abstract

Atherosclerosis (AS) is a disease characterized by the buildup of fat and fibrous elements within the walls of arteries and is a primary factor in the occurrence of heart failure and mortality. The potential targets and mechanisms underlying the anti-atherosclerotic effects of avenanthramide (Avn) were investigated using network pharmacology, molecular docking, and molecular dynamics simulations. Target information for Avn A, B, and C was collected from the PubChem and Swiss Target Prediction databases. Potential therapeutic targets for AS were identified by mining the OMIM, DrugBank, DisGeNET, and GeneCards databases. A protein–protein interaction (PPI) network of shared targets was constructed and visualized using the STRING database and Cytoscape 3.9.1. Gene Ontology and Kyoto Encyclopedia of Genes and Genomes analyses were conducted to explore the functions of core targets within the PPI network. Molecular docking was performed using the AutoDockTool to verify the correlation between the 3 types of Avns and the core targets. Furthermore, molecular dynamics simulations were performed using the 3 highest molecular docking binding energies to validate and confirm the binding of potent compounds to the target. The results revealed 109 respective targets for Avn, with 55 common targets identified by intersection with AS-related targets. Five pivotal genes, matrix metalloproteinase-9 (MMP9), epidermal growth factor receptor (EGFR), ICAM1, CASP3, and MMP2, were selected from the PPI network. Molecular docking results showed a strong binding affinity between Avn and MMP9 as well as EGFR. Molecular dynamics simulations showed good binding capacity of Avn A, B, and C with EGFR, validating the reliability of the molecular docking results. Avn potentially exerts its effects through multiple targets and displays anti-inflammatory and anti-oxidative stress properties.

## 1. Introduction

Atherosclerosis (AS) is a widespread chronic inflammatory disease of the arterial wall that often leads to disability and even death.^[1]^ AS is the leading cause of cardiovascular disease (CVD), including myocardial infarction, heart failure, stroke, and claudication.^[2]^ AS is a complex chronic disease that involves the deposition of lipids and cholesterol within the arterial wall, leading to progressive thickening and plaque formation. Over time, these plaques may harden, calcify, and eventually lead to narrowing or blockage of arteries, affecting blood flow.^[1,3]^ AS is classically associated with altered lipid metabolism and hypercholesterolemia. However, disease pathogenesis appears to be more complex than changes in lipid metabolism and involves multiple factors, the most prominent of which is inflammation.^[4]^ There is strong evidence that dietary factors can influence the development of AS directly or through their effects on traditional risk factors, such as plasma lipids, blood pressure, and plasma glucose.^[5]^ Several animal studies have shown that oatmeal improves the gut microbiota and cardiometabolic risk indicators.^[6]^

The nutritional benefits of oats appear to go beyond fiber, including bioactive phytochemicals with strong anti-inflammatory effects.^[7]^ Avenanthramide (Avn) exhibits antioxidant, anti-inflammatory, and antiapoptotic properties.^[8]^ Avn A, an active ingredient derived specifically from oats, possesses antioxidant, anti-inflammatory, and anticancer activities.^[9]^ Avn C inhibits IL-1β-mediated expression and activity of matrix metalloproteinases (MMPs).^[10]^ In addition, it inhibits the IgE-stimulated secretion of inflammatory cytokines by inhibiting the FcεRI-mediated signaling proteins Lyn, Syk, Akt, and nuclear factor kappa-light-chain-enhancer of activated B cells (NF-kB).^[11]^ Partially purified Avn from oats is nontoxic to monocytes and human aortic endothelial cells.^[12]^ Antioxidant Avns present in oats have potential anti-inflammatory and antiatherogenic effects.^[13]^ However, the underlying molecular mechanisms remains unknown.

**Figure 1.**
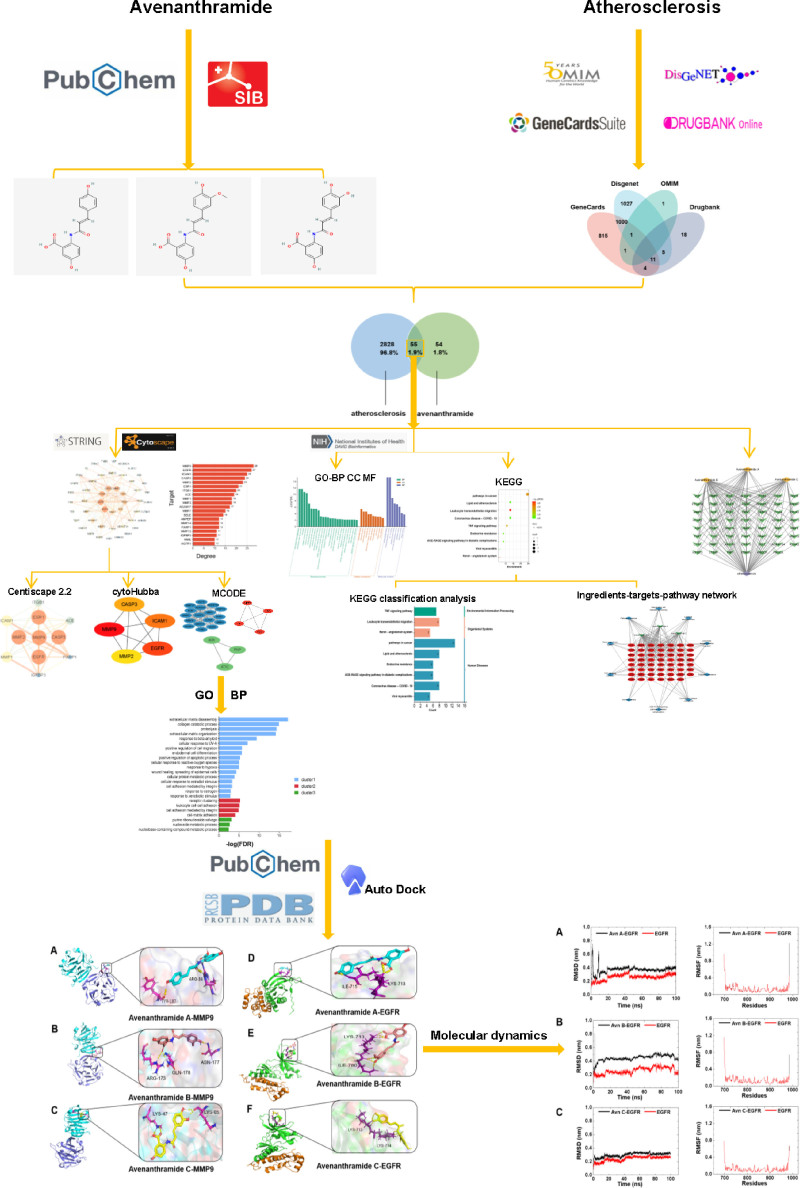
Network pharmacology and molecular docking workflow of Avn for the treatment of AS. AS = atherosclerosis, Avn = avenanthramide.

Network pharmacology and molecular docking are computational approaches that may provide insights into AS treatment. First, by integrating databases, network pharmacology can be used to identify potential biomarkers, key proteins, and metabolic pathways that are involved in disease development. Molecular docking techniques were used to identify potential targets. This helps assess whether Avn affects the biological processes (BP) associated with AS. The combined use of these methods allows the prediction of drug targets, screening of potential drug candidates, and study of the mechanisms of drug intervention. This will further our understanding of AS in the discovery and design of new therapies. The flow of the research process is summarized in Figure 1.

## 2. Materials and methods

### 2.1. Collection of Avn A, B, and C targets

First of all, the standard and Canonical SMILES of Avn A, B, and C were determined by searching for “Avenanthramide A, Avenanthramide B, and Avenanthramide C” using the PubChem database (PubChem (nih.gov)). Based on this search result, SMILES obtains possible targets from Swiss Target Prediction (Swiss Target Prediction).

### 2.2. Selection of AS-related target network

Potential therapeutic targets of AS were excavated from OMIM database (Home – OMIM), DisGeNET database (DisGeNET – a database of gene–disease associations), GeneCards database (GeneCards-Human Genes| Gene Database| Gene Search) and DrugBank database (DrugBank Online| Database for Drug and Drug Target Info) using the keyword of “Atherosclerosis.” By Venn 2.1.0 (Venny 2.1.0 (liuxiaoyuyuan.cn)) diagram to show all targets, and the targets of AS were intersected with the targets of medication, and the overlaps were selected as potential targets for AS intervention. Cytoscape 3.10.1 was applies to establish the relationship between the medication, target, and disease.

### 2.3. Construction of protein interaction network and screening of core targets

The intersecting genes representing potential targets of Avn-inhibiting AS were input into the STRING database (STRING: functional protein association networks (string-db.org)). The analysis was conducted by restricting the species to “*Homo sapiens*,” setting the “Minimum required interaction score” to “Medium Confidence > 0.4, and “Hide disconnected nodes in the network.” These parameters ensured the analysis of the target genes. Centiscape 2.2 plug-in in Cytoscape 3.9.1 were used to analyze the network. After visualization using Cytoscape 3.7.1, the network topology parameters (degree, betweenness, and closeness) of these targets were obtained using the NetworkAnalyst tool in Cytoscape. The following standards were applied to screen core targets: nodes corresponding to targets that simultaneously fulfilled the following conditions were selected as core targets for medication-input AS: betweenness > median, closeness centrality > median, and degree centrality > median. The Cyto-Hubba and MCODE plug-ins were used to further identify hub genes and potential functional modules.

### 2.4. Gene Ontology (GO) and Kyoto Encyclopedia of Gene and Genomes (KEGG) enrichment analysis

To investigate the biological functions of potential targets in Avn-inhibited AS, the DAVID database (DAVID Functional Annotation Tools (ncifcrf.gov)) was used to gather data for GO and KEGG pathway enrichment analyze. GO analysis was performed for 3 aspects: BP, molecular function, and cellular component. Then, the data were screened with a false discovery rate (FDR) ≤ 0.01 and visualized by the bioinformatics site (bioinformatics.com.cn). The FDR ≤ 0.01 KEGG pathway was classified and summarized according to the first 6 classifications in the KEGG PATHWAY Database. Pathways obviously irrelevant to AS were removed from the KEGG enrichment results. Cytoscapee 3.10.1 was used to construct an ingredient–target–pathway network.

### 2.5. Molecular docking of Avn and its core targets

Molecular docking between core components and targets was performed based on the above analysis. The SDF files of Avn A, B, and C were obtained from the PubChem database (PubChem (nih.gov)), converted to pdb files using OpenBabel, hydrogenated in AutoDock Tools, selected as ligands, and exported as pdbqt files. The 3D crystal structures of the target proteins were obtained from the Protein Data Bank (PDB) (RCSB PDB: Homepage). Water molecules and original ligands were removed from target proteins through PyMOL and subsequently imported to AutoDock Tools 1.5.6 for hydrogenation, charge calculation, and nonpolar hydrogen combination. After determining the Grid Box size and genetic algorithm, we ran AutoDock for molecular docking. Finally, PyMOL was used to visualize the results.

### 2.6. Molecular dynamics simulations

Molecular dynamics simulations of the protein–ligand complexes, derived from molecular docking, was conducted using GROMACS (version 2022.4). The protein residues and ligand topology were determined using pdbficer, sobtob, and Amber-14ffsb force fields. Prior to the simulation, the TIP3P explicit water model was employed to hydrate the system, and neutrality of the system was achieved by adding the sodium and chloride ions. Subsequently, the steepest descent method was applied, with up to 5000 iterations, to optimize the energy of the dissolve system. The simulation was set to maintain a temperature of 300 K for a constant number of atoms, volume, and temperature (NVT) ensemble, and a pressure of 1.0 bar for a constant number of atoms, pressure, and temperature (NPT) ensemble. Ultimately, 100 ns molecular dynamic simulations were executed constraints, employing a leap-frog integrator with a time step of 2 fs. The root mean square deviation (RMSD) and the root mean square fluctuation (RMSF) were analyzed.

## 3. Results

### 3.1. The targets of Avn and AS

**Figure 2.**
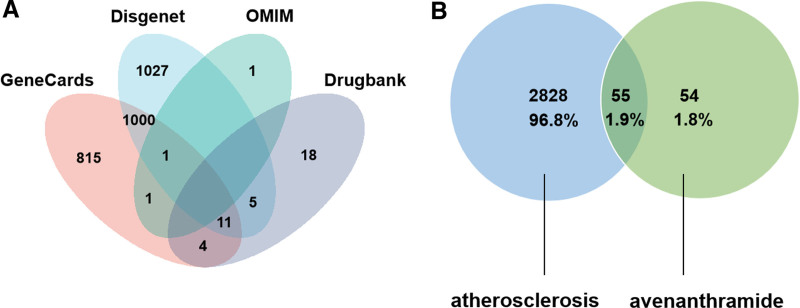
(A) Venn diagram of AS-related targets in 4 disease databases. (B) The intersection genes of identified AS targets and targets of Avn A, B, and C. AS = atherosclerosis, Avn = avenanthramide.

In total, the structures of Avn A, B, and C were obtained (Table S1, Supplemental Digital Content, http://links.lww.com/MD/O207). We initially screened 109 targets from both the PubChem and Swiss Target prediction databases, and 2883 targets were determined using the GeneCards, OMIM, DisGeNET, and DrugBank databases after removing repetitive targets (Fig. 2A). A total of 55 intersecting targets were obtained, which acted as potential targets for the inhibition of AS by Avn. The Venn diagram illustrates the targets of Avn and AS (Fig. 2B).

**Figure 3.**
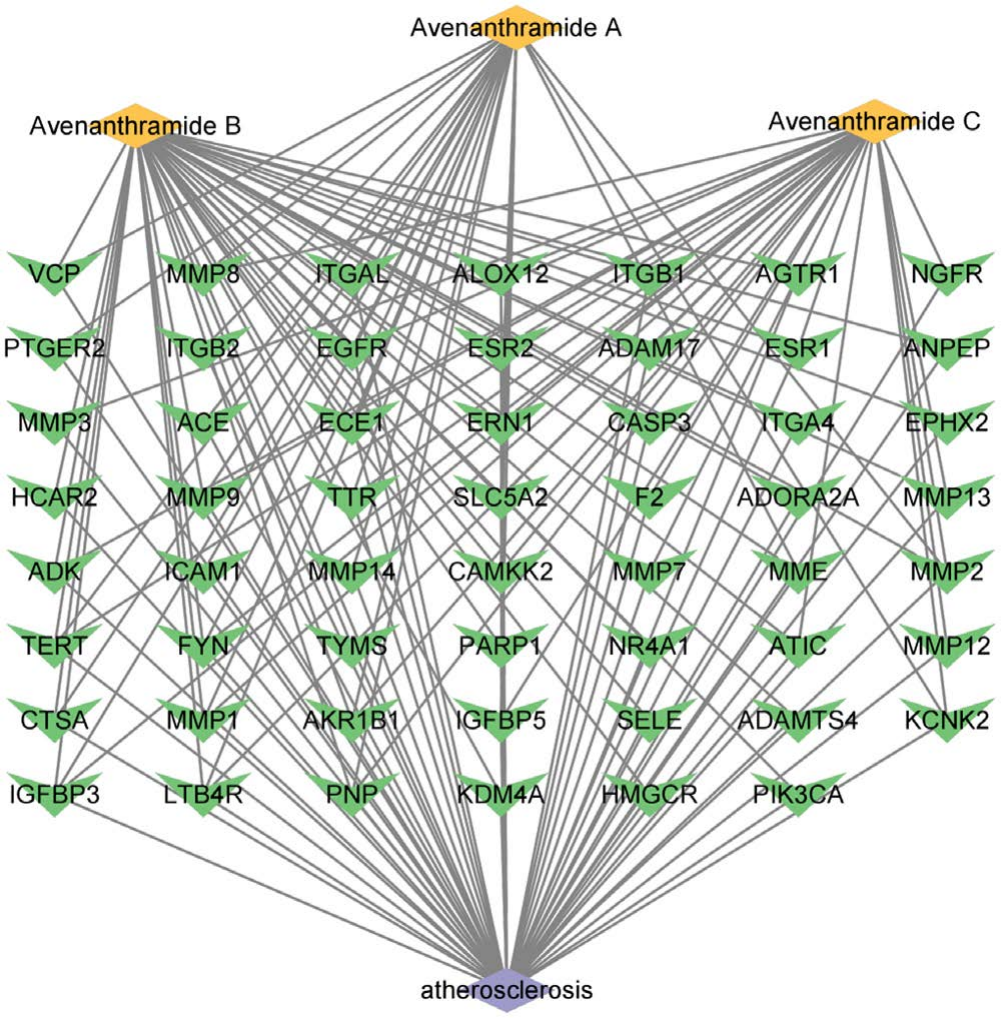
The network of the relationship between AS and the targets of Avn. AS = atherosclerosis, Avn = avenanthramide.

Intersection was conducted among the 109 targets of 3 kinds of Avn screened from 2 databases, and 2883 targets were found to be highly relevant to AS through analysis of 4 databases. Notably, the overlapping regions between these 2 datasets revealed a set of 55 potential targets that were specifically associated with Avn-inhibiting AS. Cytoscape 3.10.1 was used to build a network of medical target–diseases. Yellow, green, and purple rhombuses represent medication, target points, and disease, respectively. This network of 58 nodes and 97 edges indicated the existence of multiple interactions among various points regarding the therapeutic effect of Avn on AS (Fig. 3).

### 3.2. Construction and analysis of protein interaction

**Figure 4.**
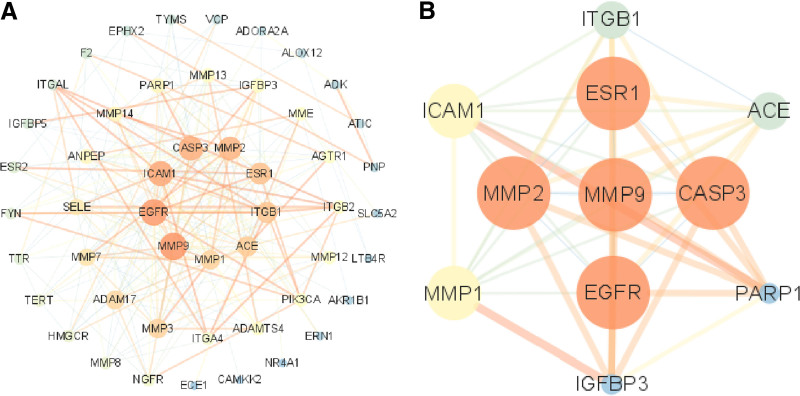
(A) The PPI network of intersection targets. (B) Core targets PPI network screened from (A) in Cytoscape 3.7.1. Deeper node color indicate higher degree values. PPI = protein–protein interaction.

Fifty-five common targets were identified between AS targets and Avn. Using String 12.0, a protein–protein interaction (PPI) network containing 50 nodes and 254 edges was constructed (Fig. 4A). The node size and color correspond to their respective degree values, with larger and more vibrant nodes representing a higher degree. Edge thickness and darkness were proportional to connectivity scores. From the network analysis, a set of 11 core targets for Avn-inhibited AS was identified, and a PPI network diagram was created, specifically showing the interaction among these core targets (Fig. 4B).

**Figure 5.**
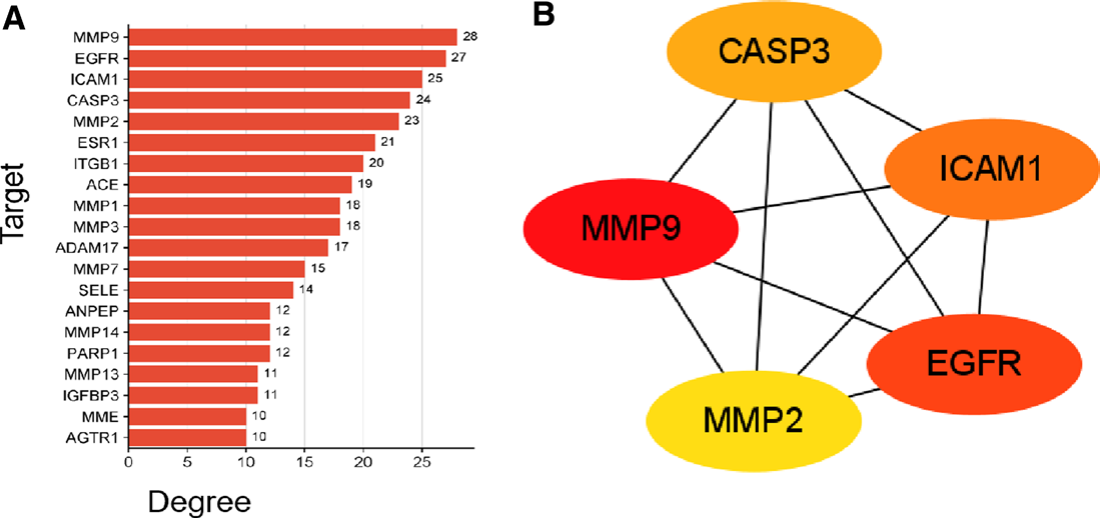
(A) Bar chart of intersection targets with top 20 degree values. (B) The top 5 hub genes.

Figure 5A shows the top 20 targets ranked by degree. Notably, the top 2 targets, based on their degree values, were 67 kDa MMP9 and epidermal growth factor receptor (EGFR). The topological parameters of the core target network are listed in Table 1. The top five hub genes (MMP9, EGFR, ICAM1, CASP3, and MMP2) were identified by the Cyto-Hubba plug-in using MCC algorithm (Fig. 5B).

**Table 1:** The core of target with degree betweenness and closeness.

Target	Degree	Betweeness	Closeness
MMP9	28	210.0460172	0.013513514
EGFR	27	287.3569338	0.01369863
ICAM1	25	181.6265731	0.012987013
CASP3	24	274.8412542	0.013157895
MMP2	23	107.4192976	0.012658228
ESR1	21	250.2166295	0.012658228
ITGB1	20	76.37870677	0.011904762
ACE	19	271.3078131	0.011764706
MMP1	18	64.20280142	0.011764706
PARP1	12	90.96698112	0.010204082
IGFBP3	11	70.50535505	0.00990099

EGFR = epidermal growth factor receptor, MMP9 = matrix metalloproteinase-9.

**Figure 6.**
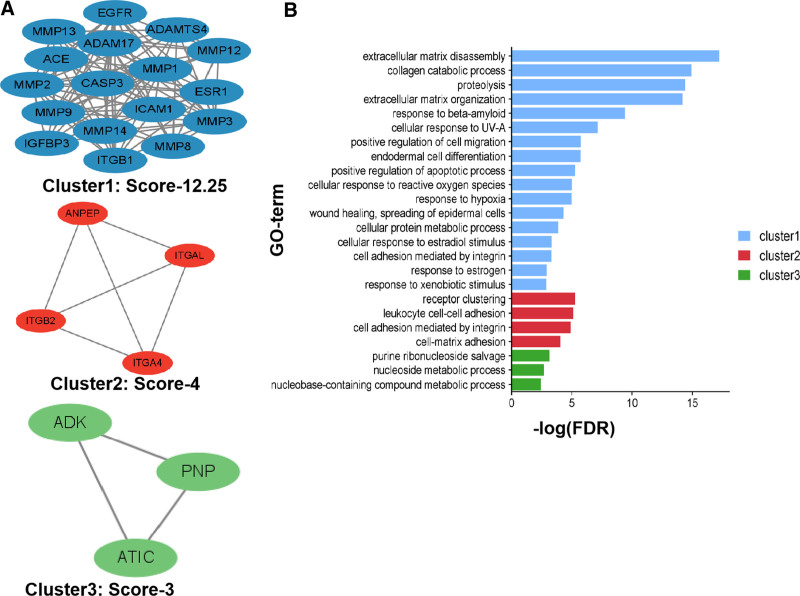
(A) GO-BP analysis of the 3 protein clusters. (B) The 3 protein clusters obtained in the MCODE plug-in analysis. BP = biological process, GO = Gene Ontology.

Protein clustering of the targets was performed using the MCODE plug-in and 3 different protein clusters were obtained (Fig. 6A). The protein clusters were subjected to GO-BP analysis. Cluster 1, based on the MMP9 protein, is involved in extracellular matrix disassembly, collagen catabolism, proteolysis, extracellular matrix organization, response to beta-amyloid, cellular response to UV-A, positive regulation of cell migration, and endodermal cell differentiation. Cluster 2, based on the seed of the ITGA4 protein, was involved in receptor clustering, leukocyte cell–cell adhesion, cell adhesion mediated by integrin, cell–matrix adhesion, and integrin-mediated signaling pathways. Cluster 3, based on the seed of the ATIC protein, was involved in purine ribonucleoside salvage, nucleoside metabolic processes, and nucleobase-containing compound metabolic processes (Fig. 6B).

### 3.3. GO and KEGG functional enrichment analysis

**Figure 7.**
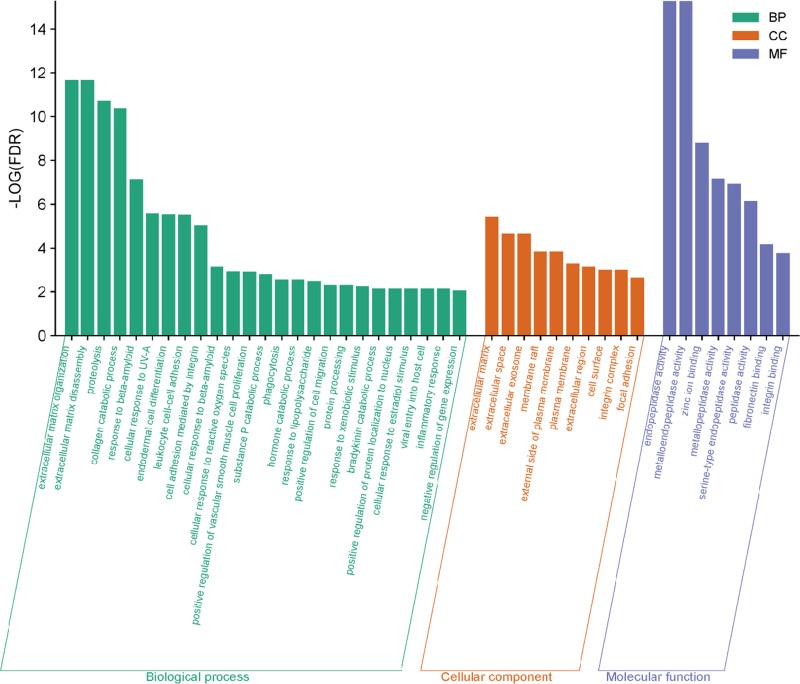
GO enrichment analysis (the FDR < 0.01 terms of BP, CC, and MF enrichment analysis were shown in green, orange, and purple bars respectively). BP = biological process, CC = cell component, FDR = false discovery rate, GO = Gene Ontology, MF = molecular function.

We performed GO analysis of the 55 potential targets using the DAVID database, limiting the species to *H sapiens*. Our analysis yielded 210 statistically significant GO entries, including 141 BPs, such as extracellular matrix organization, extracellular matrix disassembly, proteolysis, collagen catabolic process, response to beta-amyloid, and cellular response to UV-A. Twenty-six cellular components, including the extracellular matrix, extracellular space, extracellular exosome, and membrane raft; and 43 molecular functions, including endopeptidase activity, metalloendopeptidase activity, zinc ion binding, and metallopeptidase activity. The GO terms were ranked based on FDR values, and for FDR < 0.01 the terms were selected and visually depicted in the enrichment analysis diagram (Fig. 7).

**Figure 8.**
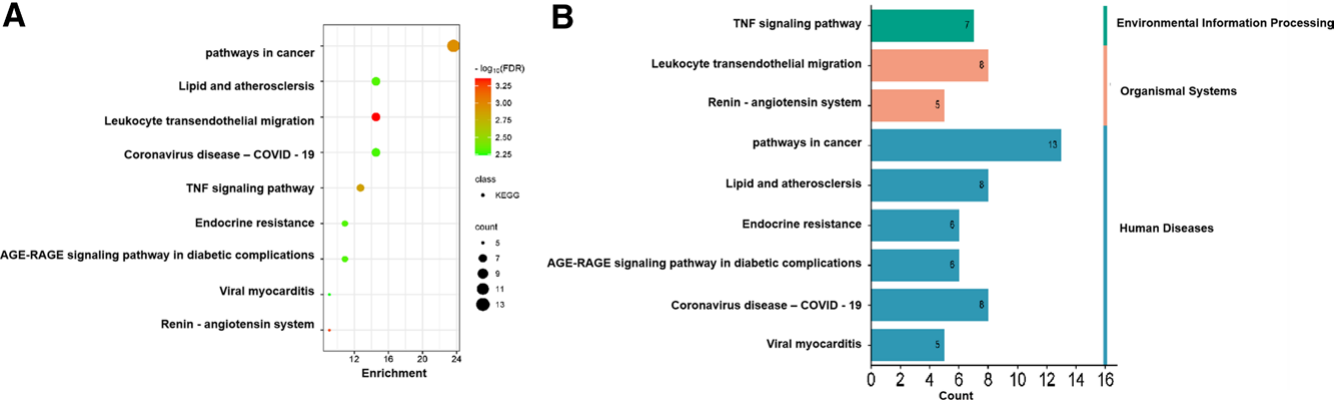
(A) Bar graph of KEGG enrichment analysis for 55 intersecting targets (FDR < 0.01 results). (B) Taxonomic analysis of KEGG enrichment pathways. KEGG = Kyoto Encyclopedia of Genes and Genomes.

KEGG analysis was also conducted on these 55 potential targets using the DAVID database to identify their involvement in specific signaling pathways. Among the 51 enriched signaling pathways, we generated a statistical bubble chart and categorical data. Avn A, B, and C were majorly involved in pathways in cancer, lipid and AS, leukocyte transendothelial migration, and the tumor necrosis factor (TNF) signaling pathway (Fig. 8A and Table 2), illustrating the frequency and significance of the enrichment score and the level of significance, with taller bar counts and higher enrichment. It presented a concise and visually informative depiction of the top 20 enriched KEGG signaling pathways, emphasizing the pathway that was particularly relevant to Avn inhibition of AS (Fig. 8B).

**Table 2:** The pathway of FDR < 0.01 associate with AS.

Class	Description	Enrichment	Count	Geen ID
KEGG	Leukocyte transendothelial migration	14.54545455	8	ITGB1, PIK3CA, ITGA4, MMP2, ITGB2, ITGAL, MMP9, ICAM1
KEGG	Renin–angiotensin system	9.090909091	5	CTSA, ACE, MME, ANPEP, AGTR1
KEGG	Pathways in cancer	23.63636364	13	ITGB1, MMP1, PTGER2, MMP2, F2, ESR1, MMP9, EGFR, ESR2, TERT, PIK3CA, CASP3, AGTR1
KEGG	TNF signaling pathway	12.72727273	7	MMP14, PIK3CA, CASP3, MMP3, SELE, MMP9, ICAM1
KEGG	Lipid and atherosclerosis	14.54545455	8	ERN1, PIK3CA, MMP1, CASP3, MMP3, SELE, MMP9, ICAM1
KEGG	Endocrine resistance	10.90909091	6	PIK3CA, MMP2, ESR1, MMP9, EGFR, ESR2
KEGG	AGE–RAGE signaling pathway in diabetic complications	10.90909091	6	PIK3CA, CASP3, MMP2, AGTR1, SELE, ICAM1
KEGG	Coronavirus disease (COVID-19)	14.54545455	8	ADAM17, ACE, PIK3CA, MMP1, MMP3, AGTR1, F2, EGFR
KEGG	Viral myocarditis	9.090909091	5	CASP3, ITGB2, FYN, ITGAL, ICAM1

AS = atherosclerosis, FDR = false discovery rate, KEGG = Kyoto Encyclopedia of Gene and Genomes, TNF = tumor necrosis factor.

**Figure 9.**
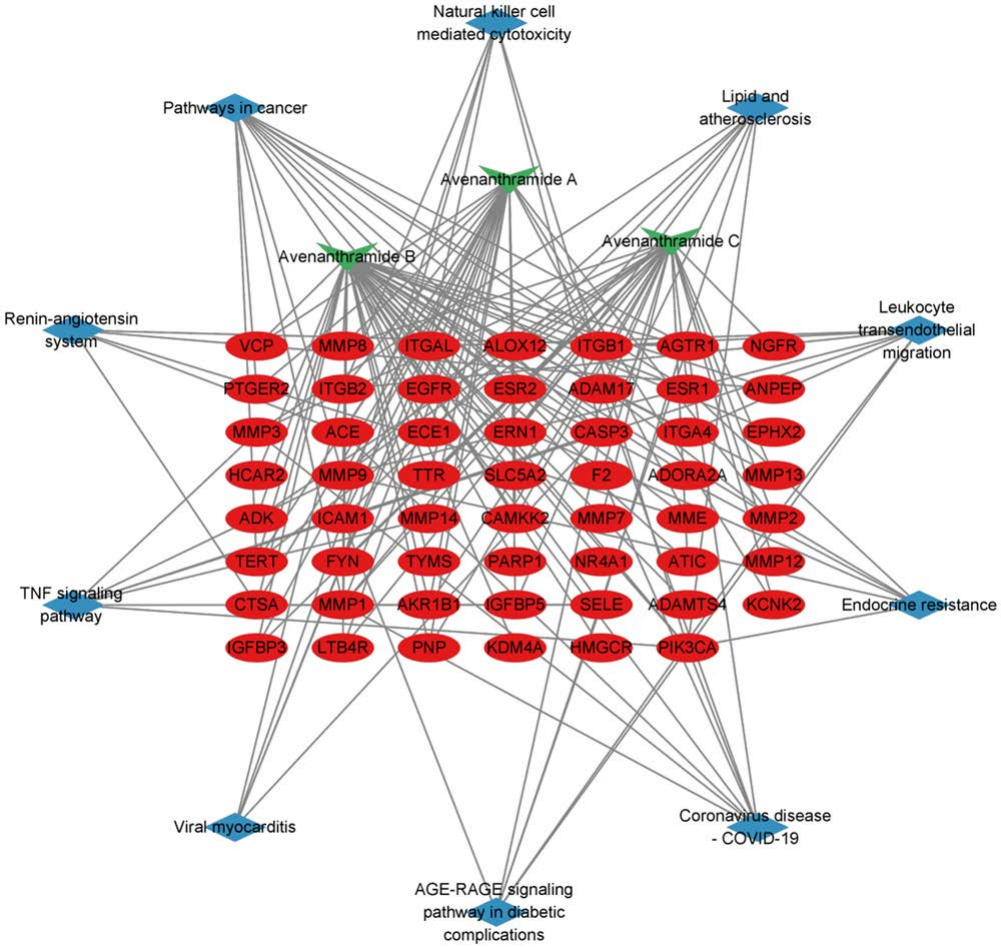
Ingredient–target–pathway network.

The ingredient–targets–pathway network were built with the AS-related pathway (Fig. 9), in which MMP9, CASP3, MMP2, ICAM1, and EGFR target genes were top ranked. Each pathway interacts with common targets, indicating that Avn could treat AS by orchestrating multiple pathways.

### 3.4. Molecular docking

**Figure 10.**
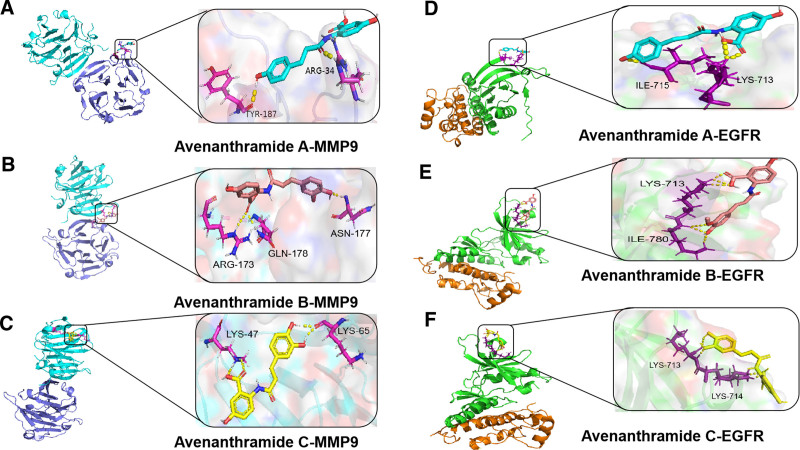
(A) Molecular docking results of Avn A and MMP9 (affinity: -4.42). (B) Molecular docking results of Avn B and MMP9 (affinity: -4.42). (C) Molecular docking results of Avn C and MMP9 (affinity: -4.03). (D) Molecular docking results of Avn A and EGFR (affinity: -5.85). (E) Molecular docking results of Avn B and EGFR (affinity: -6.30). (F) Molecular docking results of Avn C and EGFR (affinity: -5.58). Avn = avenanthramide, EGFR = epidermal growth factor receptor, MMP9 = matrix metalloproteinase-9.

Molecular docking analysis was conducted to investigate the interactions between Avn A, B, and C and 2 core target genes (MMP9 and EGFR) (Table 3). All 6 docking results were generated using the AutoDock software and displayed low binding energies, indicating strong affinities between the compound and targets. Notably, all 5 core target proteins exhibited robust binding to MMP9 and EGFR with binding energies of <0, indicating that Avn and each core target can bind spontaneously, signifying their significant roles in the molecular mechanism by which Avn inhibits AS. The lowest binding energies between each target were visualized using PyMOL (Fig. 10).

**Table 3:** Molecular docking of core target and Avn A, B, and C.

Protein	PDB ID	Docking compound	Docking result	Affinity
MMP9	1ITV	Avn A	TYR-189 ARG-34	-3.08
		Avn B	ARG-173 GLN-178 ASN-177	-2.42
		Avn C	LYS-47 LYS-65	-4.03
EGFR	5GNK	Avn A	LYS-713 ILE-715	-5.81
		Avn B	LYS-713 ILE-780	-6.30
		Avn C	LYS-714 LY713	-5.58

Avn = avenanthramide, EGFR = epidermal growth factor receptor, MMP9 = matrix metalloproteinase-9.

### 3.5. Molecular dynamics validation

**Figure 11.**
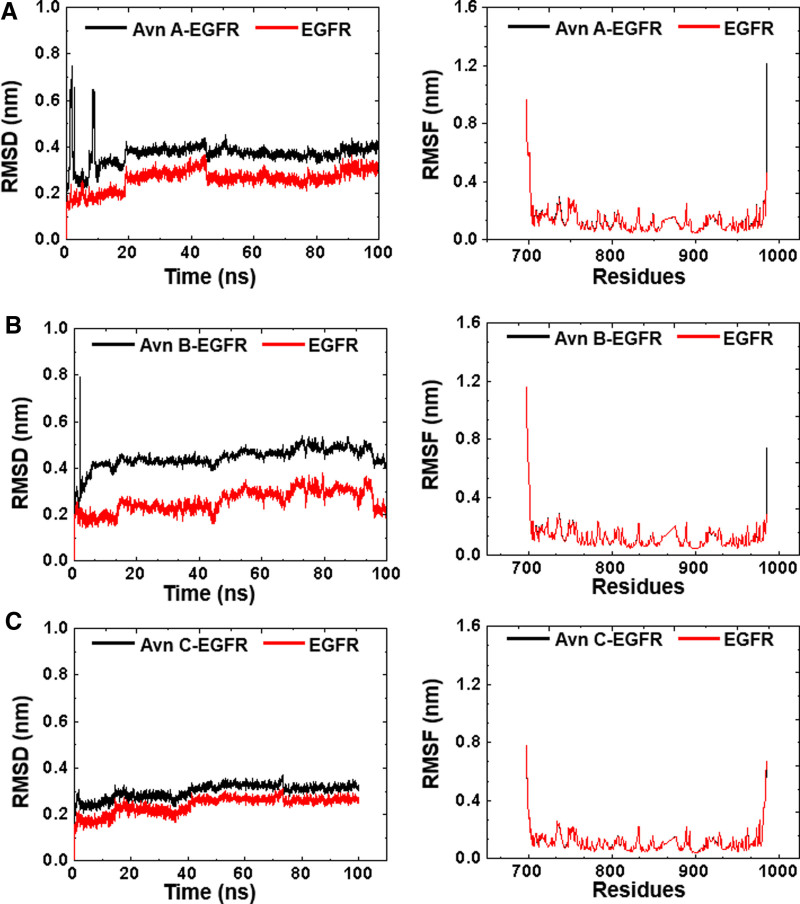
The results of molecular dynamics simulations. (A) RMSD and RMSF values for EGFR protein and its complexes with Avn A. (B) RMSD and RMSF values for EGFR protein and its complexes with Avn B. (C) RMSD and RMSF values for EGFR protein and its complexes with Avn C. Avn = avenanthramide, EGFR = epidermal growth factor receptor, RMSD = root mean square deviation, RMSF = root mean square fluctuation.

Subsequently, molecular dynamics simulations were performed to verify the binding abilities of small molecular compounds and key target proteins with optimal binding abilities in molecular docking. Avn A, B, and C and EGFR protein (5gnk) were chosen for molecular dynamics simulations. Since molecular docking studies demonstrated superior binding energies compared to MMP9, these compounds were selected for further evaluation of their binding affinities to key target proteins through molecular dynamics simulations. The free EGFR protein and its complex with Avn A began to stabilize at 45 ns, and the value of EGFR protein fluctuated from 0.2 to 0.3 nm, while that of protein complex fluctuated from 0.3 to 0.4 nm (Fig. 11A). The RMSD value of EGFR was slightly increased upon binding of Avn B, from 0.2 to 0.45 nm, but the complex was stabilized after about 20 ns and remain stable until the end of simulation (Fig. 11B). The RMSD value of the free EGFR protein and its complex with Avn C remained stable. Furthermore, there were no significant residue fluctuations for the EGFR protein due to the binding of Avn A, B, and C (Fig. 11C). These findings demonstrated the existence of stable interactions between the 3 molecules and the EGFR protein (Fig. 11A–C), subsequently bolstering the reliability of molecular docking predictions.

## 4. Discussion

CVD has surpassed malignant tumors as the leading cause of mortality, with AS playing a significant role as a pathological factor.^[14]^ Dysfunction of endothelial cells lining susceptible regions of the arterial vasculature significantly contributes to AS in atherosclerotic CVD.^[15]^ Numerous factors act as inducers of the inflammatory process in AS, including vascular endothelium aging, metabolic dysfunction, autoimmune diseases, and infectious damage factors.^[16]^ Furthermore, chronic kidney disease accelerates AS via augmentation of inflammation, perturbation of lipid metabolism, and other mechanisms. In the arterial wall, the subendothelial retention of plasma lipoproteins triggers monocyte-derived macrophages and T helper type 1 cells to form atherosclerotic plaques.^[17]^ Inhibition of miR-33 using pHLIP-directed macrophage targeting improves AS regression by increasing collagen content and decreasing lipid accumulation within the vascular lesions.^[18]^

In related studies, Avn C protected normal human skin fibroblasts against oxidative stress and inflammatory responses through NF-κB inhibition and Nrf2/HO-1 activation.^[19]^ The existing literature indicates that a high Avn has demonstrable beneficial effects in the prevention of CVDs.^[20]^ In this study, we found that the primary targets of 3 types of Avn were MMP9 and EGFR. Some studies have already shown that MMP9 plays a major role in the pathogenesis of AS and restenosis by regulating both migration and proliferation of vascular smooth muscle cells after arterial injury,^[21]^ and that gene deletion of EGFR in myeloid cells limits IL-6 and TNF-α production, lipid uptake, and consecutively reduces AS development.^[22]^

GO functional analysis revealed that 3 kinds of Avn play a therapeutic role in AS by participating in and regulating BPs such as extracellular matrix organization, extracellular matrix, and endopeptidase activity. KEGG pathway analysis inferred that the main signaling pathways of Avn intervention in AS were cancer, lipid and AS, leukocyte transendothelial migration, and TNF signaling.

As for leukocyte transendothelial migration, during inflammation, endothelial cells are activated by inflammatory cytokines to express adhesion molecules and synthesize chemo-kines and lipid chemoattractants that are presented on their luminal surface. Activated endothelial cells also transport chemoattractants from their abluminal surface 45 other chemoattractants can be generated by proteolytic cleavage in activated mast cells and platelets and delivered to endothelial cells through circulating microparticles or exocytosis of intracellular granules.^[23]^ One of the causes of AS is the inflammatory response, which can activate the pathway of leukocyte transendothelial migration, thereby achieving a therapeutic effect in treating AS. The lipid and AS pathways contain 8 targets. It mainly involves the phosphoinositide 3-kinase, AKT, TNF, c-Jun N-terminal kinase, and mitogen-activated protein kinase (MAPK) 1/3 pathways, and its functions are mainly focused on the proinflammatory, inflammatory signaling cytokines, proliferation, injury, and apoptosis processes.^[24]^ Studies have shown that activation of the phosphoinositide 3-kinase/AKT signaling pathway reduces the expression of TLR4, which is critical for foam cell formation, prevention of atherosclerotic plaque formation, and destabilization.^[25]^

It has been known that development of AS is closely related to activation of TNF-α. This study found that inhibiting the synthesis of TNF-induced proteins could also suppress the occurrence of AS.^[26]^ The impaired NO production mentioned earlier is a significant reason for the development of AS. Avn C inhibited the expression of MMP9 and cell migration through the MAPK/NF-κB signaling pathway in TNF-α-activated HASMC. Therefore, Avn C can be identified and used as a disease-prevention material and remedy for AS.^[27]^

Molecular docking results indicated that the docking energies between Avn A, B, and C, MMP9, and EGFR were all <0, suggesting a strong binding interaction between them. Molecular dynamics simulation studies provide strong evidence for this result. This indicates that Avn A, B, and C may collectively regulate AS through multi-target interactions.

Despite encouraging discoveries in the mechanisms of Avn A, B, and C against AS, there are still certain limitations. First, this study was largely based on existing databases, which, if not sufficiently comprehensive and scrutinized, may generate insufficient inferences, and the combined role of these 3 active ingredients in activating or inhibiting ad-related pathways is unknown. Moreover, the results of computational chemistry may not be consistent with the actual therapeutic effect, and need to be further confirmed by in vivo and in vitro experiments.

## 5. Conclusions

Based on network pharmacology and molecular docking, this study revealed the key targets of Avn A, B, and C intervention in AS, as well as the main signaling pathways (lipid and AS, leukocyte transendothelial migration, and TNF signaling pathway). Avn A, B, and C can regulate different targets, and the same target can interfere with different BPs and signaling pathways, showing the characteristics of multi-target and multi-pathway interventions.

## Author contributions

**Conceptualization:** Yibei Zhan.

**Data curation:** Longzhi Fang, Meng Han.

**Formal analysis:** Yuanmei Zheng.

**Funding acquisition:** Yibei Zhan.

**Investigation:** Meng Han.

**Methodology:** Longzhi Fang, Meng Han.

**Project administration:** Zhigang Wang.

**Software:** Longzhi Fang.

**Supervision:** Kangzhe Liu.

**Validation:** Kangzhe Liu.

**Visualization:** Yuanmei Zheng.

**Writing – original draft:** Zhigang Wang, Longzhi Fang, Yibei Zhan.

**Writing – review & editing:** Zhigang Wang, Longzhi Fang, Yibei Zhan.

## Supplementary Material


