# Supplementary material for: Exploring the mechanism of avenanthramide in the treatment of atherosclerosis based on network pharmacology and molecular docking: An observational study
Source: Medicine (Baltimore). 2024 Dec 20;103(51):e40932. doi: 10.1097/MD.0000000000040932 (PMC11666176; doi:10.1097/MD.0000000000040932)
Supplement: Supplementary file 1 [file medi-103-e40932-s001.docx]

**Table S1.** The structure of Avn A B and C.

| PubChem ID | Name | Canonical SMILES | Structure |
| --- | --- | --- | --- |
| 11723200 | Avenanthramide **A** | C1=CC(=CC=C1C=CC(=O)NC2=C(C=C(C=C2)O)C(=O)O)O | 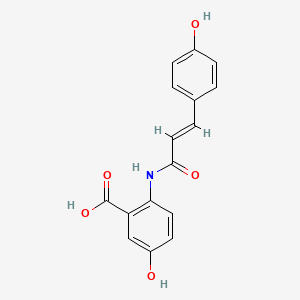 |
| 10087955 | Avenanthramide **B** | COC1=C(C=CC(=C1)C=CC(=O)NC2=C(C=C(C=C2)O)C(=O)O)O | 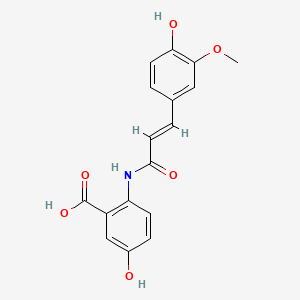 |
| 11723200 | Avenanthramide **C** | C1=CC(=C(C=C1C=CC(=O)NC2=C(C=C(C=C2)O)C(=O)O)O)O | 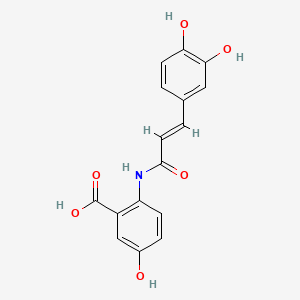 |
